# Deciphering the Structure, Growth and Assembly of Amyloid-Like Fibrils Using High-Speed Atomic Force Microscopy

**DOI:** 10.1371/journal.pone.0013240

**Published:** 2010-10-08

**Authors:** Pierre-Emmanuel Milhiet, Daisuke Yamamoto, Olivia Berthoumieu, Patrice Dosset, Christian Le Grimellec, Jean-Michel Verdier, Stéphane Marchal, Toshio Ando

**Affiliations:** 1 Inserm, U 554, Montpellier, France; 2 Université de Montpellier, CNRS, UMR 5048, Centre de Biochimie Structurale, Montpellier, France; 3 Department of Physics, Graduate School of Science and Technology, Kanazawa University, Kanazawa, Japan; 4 Inserm, U710, Montpellier, France; Université de Montpellier 2, Montpellier, France; EPHE, Paris, France; National Institutes of Health, United States of America

## Abstract

Formation of fibrillar structures of proteins that deposit into aggregates has been suggested to play a key role in various neurodegenerative diseases. However mechanisms and dynamics of fibrillization remains to be elucidated. We have previously established that lithostathine, a protein overexpressed in the pre-clinical stages of Alzheimer's disease and present in the pathognomonic lesions associated with this disease, form fibrillar aggregates after its N-terminal truncation. In this paper we visualized, using high-speed atomic force microscopy (HS-AFM), growth and assembly of lithostathine protofibrils under physiological conditions with a time resolution of one image/s. Real-time imaging highlighted a very high velocity of elongation. Formation of fibrils via protofibril lateral association and stacking was also monitored revealing a zipper-like mechanism of association. We also demonstrate that, like other amyloid ß peptides, two lithostathine protofibrils can associate to form helical fibrils. Another striking finding is the propensity of the end of a growing protofibril or fibril to associate with the edge of a second fibril, forming false branching point. Taken together this study provides new clues about fibrillization mechanism of amyloid proteins.

## Introduction

Protein aggregation and fibril formation are molecular events that have been related to the emergence of more than 20 human diseases called conformational diseases [1] or proteopathies [2] and these diseases develop with the misfolding of normally-soluble proteins. Over the past decade, significant progress has been made in understanding the structural arrangements of amyloid fibrils. Despite large differences in size, native structure and functions of proteins, numerous molecular models of amyloid fibrils display similarities [3], [4]. From a mechanistic point of view, a broad pathway of fibril formation could be schematized by one of three straightforward models: i) the “refolding” model, in which self-propagating conformational rearrangements must occur in order to adopt a structure competent for self-assembly into fibrils. That is the case for insulin [5], the SH3 domain of bovine PI3K [6], myoglobin [7] and prion proteins [8]; ii) the “natively disordered” model, composed of proteins or peptides whose native structure was mainly disordered such as polyglutamine proteins [9], huntingtin [10], ataxins, yeast prions or the amyloid beta (Aß)-peptide of amyloid plaques [11], [12]; iii) the “gain-of-interaction” model that concerns proteins having only a part of the peptide backbone involved in molecular interactions without extended structural changes of the globular portion of the protein. Direct-stacking interactions [13], cross-ß spine patterns [4], or 3D domain swapping [14], [15] governs fibril elongation in these systems.

Among the proteins classified in the latter model, lithosthatine (also named Reg-1) is a protein of 144 amino acids that is produced by pancreas acinar cells and secreted into pancreatic juice. The protein tightly binds calcium carbonate crystals and may control their formation preventing clogging of the ducts [16]–[19]. In chronic calcifying pancreatitis, the protein forms deposits in pancreatic ducts [20]. Lithostathine is also expressed in other tissues including gastric cells [21] and the protein functions as a mitogenic and/or antiapoptotic factor in the development of early gastric cancer [22]. More recently, potential role in normal and neoplastic germ cell proliferation has been described [23]. Lithostathine is very susceptible to self-proteolysis under specific pH conditions and cleavage produces a soluble N-terminal undecapeptide and a C-terminal form of 133 amino acids. This processed protein, called S1, precipitates and forms protease-K-resistant fibrils at physiological pH [24] that deposit in the brain of patients with Creutzfeldt-Jakob, Gertsmann-Straüssler-Scheinker diseases [25] and AD, especially during the very early stages [26]. On the basis of X-ray structure of the monomer [27], [28], biochemical experiments, high-resolution electron microscopy (EM), and atomic force microscopy (AFM), it was proposed that lithostathine is first assembled via lateral hydrophobic interactions as a tetramer. Each tetramer can then interact with another tetramer through electrostatic interactions to form helical structure named protofibril [26]. Structural assembly of these protofibrils can form a two-dimensional network. Recent works also suggest that juxtaposed protein units exchange a mobile loop (domain swapping) to form highly ordered fibrillar structures [25] and no cross-ß pattern was observed for fibrillar lithosthathine. Similarly to PrP^sc^, lithostathine deposits were resistant to the drastic proteinase K treatment [25].

In this paper, we present real time visualization of lithostathine fibrillization using high-speed Atomic Force Microscopy (HS-AFM) that allows for high imaging rate with good lateral and vertical resolution [29]. Under these conditions, we were able to visualize elongation of lithostathine protofibrils and their assembly into fibrils. Dynamics and remodelling of these assemblies were also observed.

## Results

### AFM imaging of the lithostathine S1 form in air

**Figure 1:**
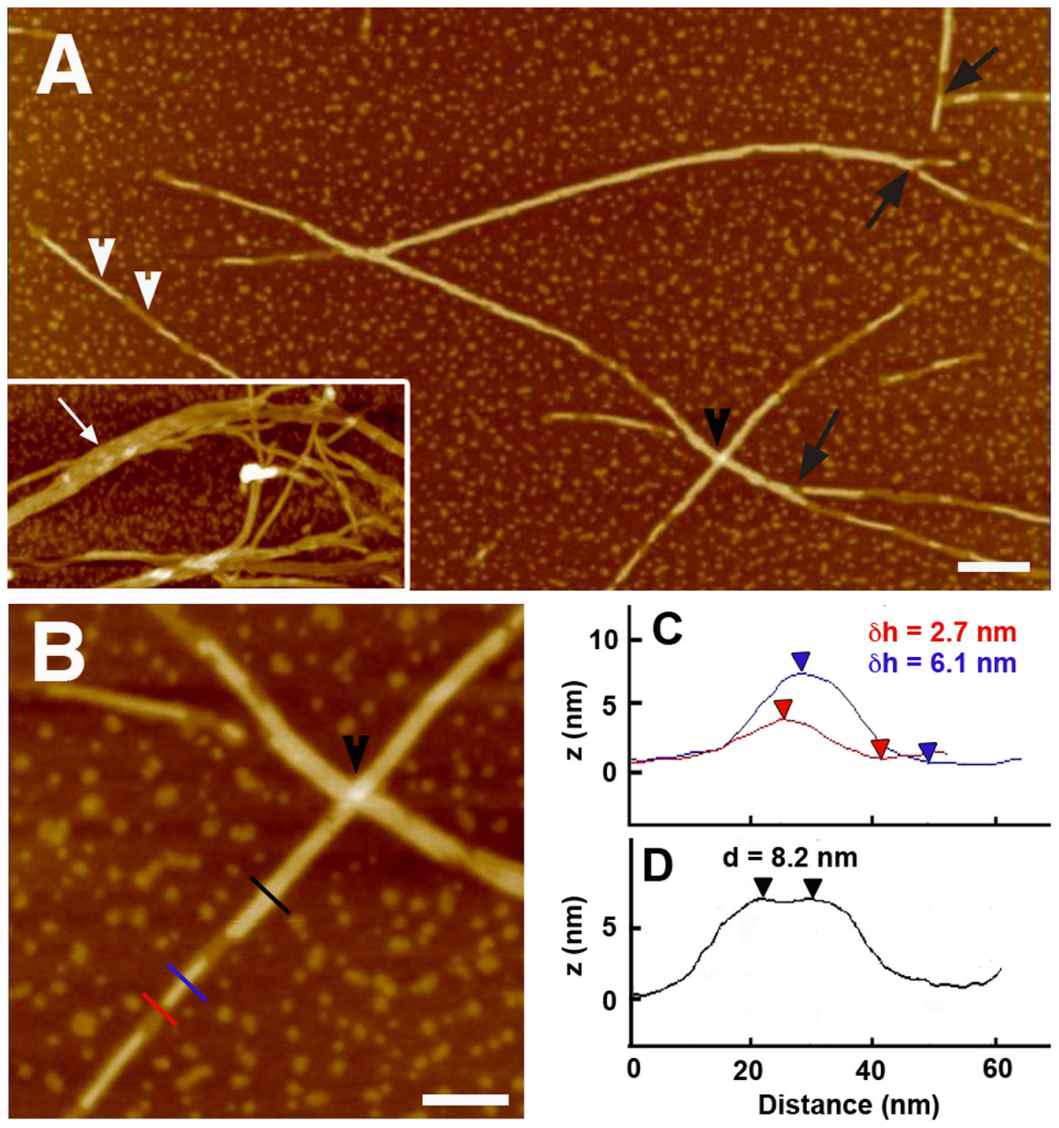
AFM height images of lithostathine in air. A and B (zoom of A) are height AFM images showing the S1 form of lithostathine forming protofibrils, isolated or laterally associated. C and D are profiles of B indicated by thin colored lines. The profile C measures the two different heights that were observed along protofibrils (white arrowheads in A) and section D the distance between two laterally associated protofibrils (top-to-top distance). The inset in A illustrates propensity of lithostathine protofibrils to form bundles on mica (white arrows). The black arrows indicate what looks like a branching point of protofibrils whereas the black arrowheads indicate overlapping filaments. The scale bars are 200 nm (A) and 100 nm (B) and the height color scale is 20 nm.

The S1 form of lithostathine was generated in solution by trypsin digestion of full-length protein that mimics native auto-maturation of the protein [24], coated on mica, and imaged using tapping mode AFM in air. As expected from a previous study [26], a network of protofibrils was observed (Fig. 1). Protofibril lengths ranged from a few tens of nanometers to several micrometers and their density was variable (see a high density in A inset). They tended to laterally associate forming bundles containing up to 10 protofibrils (white arrows in A inset). The apparent diameter of protofibril (8.7±1.1 nm) was evaluated by measuring the top-to-top distance between two adjacent protofibrils because this distance is not affected by tip convolution (see sections in Fig. 1D). This value is in the same range than that obtained by EM and molecular modeling of lithostathine tetramer [26]. The height of most of the filamentous structures as compared to mica was estimated to be 5.97±0.71 nm. However a lower height (3.04±0.52 nm), half of the first value, can be clearly identified in several cases (see white arrowheads in Fig. 1A and section in Fig. 1C). This result suggests that two protofibrils could be vertically stacked on top of each other and the height of one protofibril imaged in air could be roughly estimated to be 3 nm. AFM pictures also suggested protofibril branching (black arrows in Fig. 1A) but, under these conditions, it was difficult to discriminate between a real branching and an interaction between two protofibrils. In addition overlapping of fibrillar structures were clearly delineated by the AFM tip (black arrowheads in Fig. 1A and 1B). Interestingly globular structures were observed on mica with a size ranging from 6 to a few tens of nanometers with an apparent height similar to that measured for a protofibril.

### Structure and dynamics of lithostathine using high-speed AFM

In order to avoid damaging of the protofibrils, we then decided to perform AFM imaging in buffer, a physiological condition that minimizes the force applied by the tip. Taking advantage of the development of HS-AFM allowing data acquisition at video rates [29], we also investigated the dynamics of protofibril and fibril formation. Starting from intact lithostathine, protofibril formation was monitored in real time after addition of trypsin to the medium bathing the tip. Even though the scanning area of such a microscope is well below standard AFM, the high-speed scanner was sufficient to visualize most of the structures described above.

**Figure 2:**
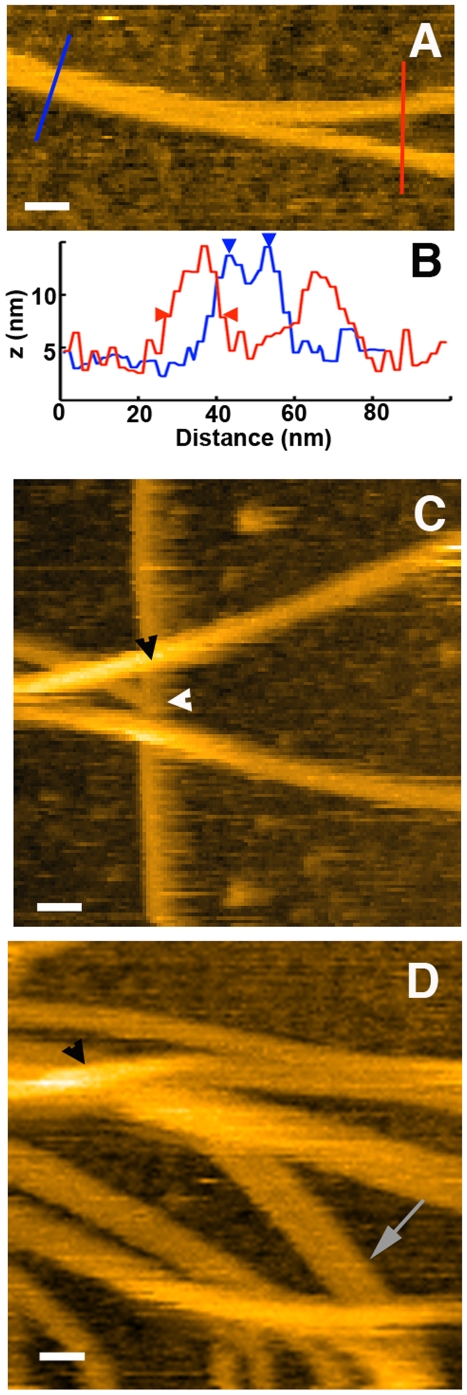
Height images of lithostathine protofibrils and fibrils using high-speed AFM. A is the height image of two partially associated protofibrils. The profile in B corresponding to the thin colored lines in A shows mid-height diameter of 16.7 nm for a single protofibril (in red, delineated by arrowheads) and a top-to-top distance of 9.7 nm for two laterally associated protofibrils (in blue). Associations of the end of one fibril with the edge of another (white arrowhead in C and Movie S1) as well as overlapping (black arrowheads in C and D) of fibrils or protofibrils were easily identified. Long incubation time with trypsin led to a complex network of protofibrils and fibrils (D) and bundles of lithosthathine protofibrils are frequently observed (grey arrow in D). The scale bars are 30 nm and the height color scale is automatically adjusted during imaging.

**Figure 3:**
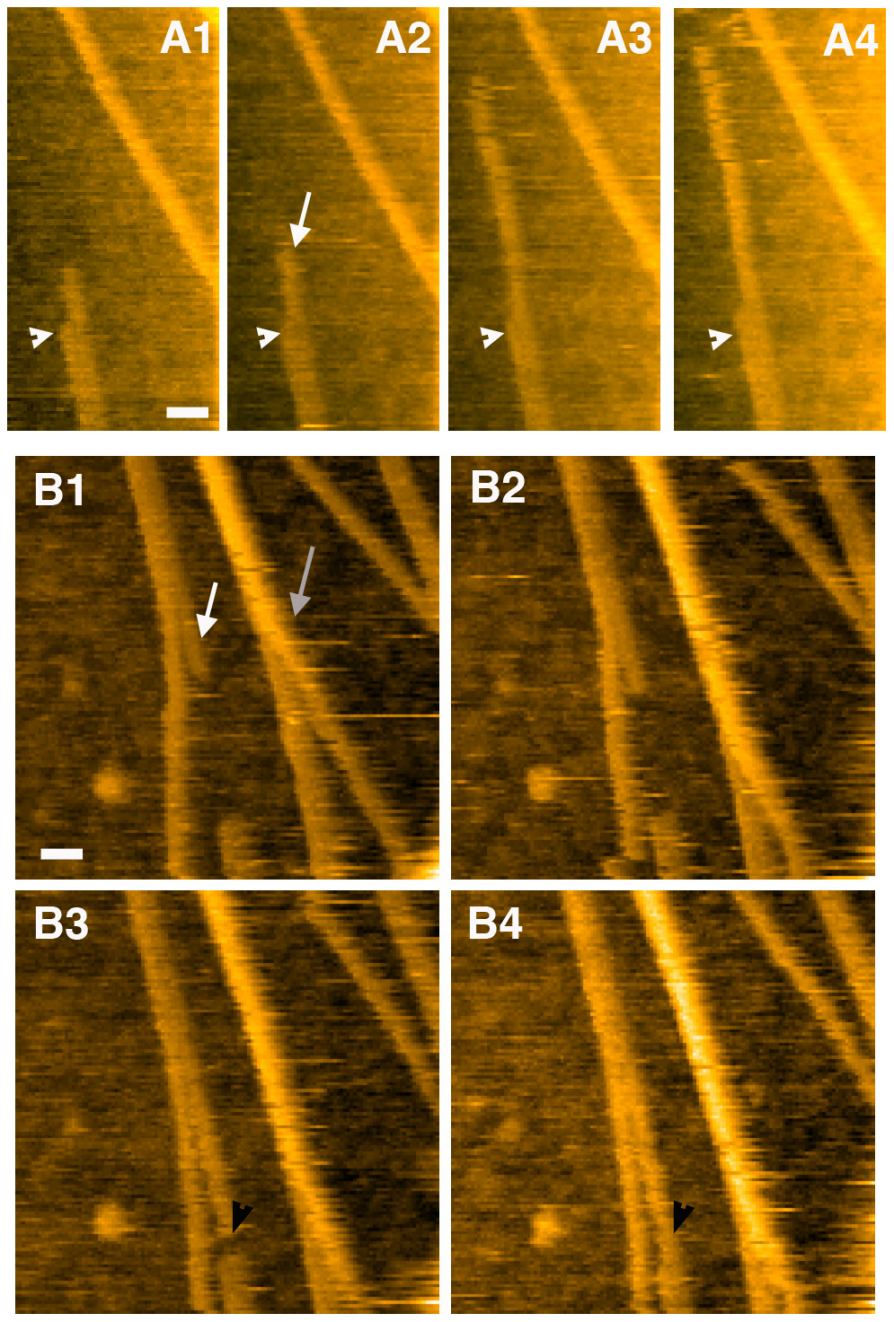
Time lapse of protofibril association and elongation. Elongation of single protofibril (white arrows) is shown in time lapses A1–A4 and B1–B4. In both cases, protofibrils are laterally associated with another, at least for one part of their length. One protofibril can grow while the position of the associated fiber is constant (white arrowheads in time lapse A). Interestingly the edge of elongating protofibril can associate with that of a pre-existing protofibril forming a continuous structure (black arrowheads in B3 and B4). Real time vertical association of 2 protofibrils is also observed in time lapse B. They partially interact in B1 (grey arrow) and are mostly stacked on top of each other in B4. A globular structure is also imaged and can be used as fiducial mark. The time resolution is 2 sec, the scale bar is 30 nm, and the height scale is automatically adjusted.

**Figure 4:**
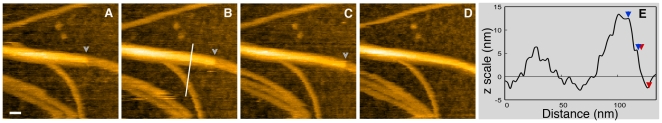
Time lapse of the protofibril elongation stacked on top of a fibril. The grey arrowheads indicate the edge of the elongating protofibril on top of a fibril. The profile in E corresponding to the white line in B indicates that only two fibers are stacked on top of each other (height differences between blue and red arrows are 6.7 and 8 nm, respectively). The underlying fibril most probably corresponds to two laterally associated protofibrils. Micrographs correspond to successive images separated by 1 s. The scale bar is 30 nm and the height color scale is automatically adjusted during imaging.

After 15 min incubation in presence of trypsin, globular structures were imaged having an apparent diameter ranging from 15 to 28 nm that compared well with our results in air (see Fig. 2, 3 and 4). These structures protruding from 6.6 to 22 nm above the mica could correspond to lithostathine oligomers (lithostathine tetramer is about 9 nm in diameter and 2.5 nm in thickness) [26]. After 30 min incubation, filamentous structures were imaged at one image per sec allowing their association as well as their elongation to be observed (see Movie S1 and snapshots in Fig. 2). The width of these structures varied largely and the thinnest filaments most probably correspond to single protofibrils (white arrows in Fig. 2A) since their apparent width measured at mid-height was 15.5±4.4 nm (Fig. 2B, red profile), a slightly overestimated value due to tip convolution as compared to the 11.7 nm obtained with EM [26]. Similarly to AFM imaging in air, lateral association of protofibrils (up to 8) was observed (Fig. 2A and D, Fig. 3A and Movie S1) and more accurate width value was obtained by measuring top-to-top distance between adjacent protofibrils (9.9±2.8 nm)(Fig. 2B, blue profile). It was sometimes difficult to visualize the groove between two adjacent protofibrils suggesting that they strongly interact along their main axis. Thanks to HS-AFM, elongation of filaments was observed in real time (see Movie S1). Both protofibrils (first part of Movie S1 and time lapse in Fig. 3) and fibrils were elongated with a velocity ranging from 27 to 52 nm/s (values correspond to velocity measured for at least 4 frames)(see Fig. S1, which corresponds to the plot of the growth distance as a function of time). The way fibers elongated in Movie S1 lead us to consider the possibility that we might be imaging a sliding process of fiber on mica rather than a real growth. However this hypothesis can be ruled out by the fact that two fibers that laterally associated can grow independently from each other (time lapse in Fig. 3A). Alternatively we might expect the tip scanning to influence a putative sliding process but such a phenomenon was never observed. Another interesting feature of elongating protofibril was their ability to connect to another pre-existing protofibril. As shown in Fig. 3 (black arrowheads in B3 and B4), the edge of the elongated protofibril fused with another to form a continuous structure (at least at this scale). In this time lapse, preferential direction of growth of the protofibril can be explained by the fact that it was interacting with another fibrillar structure that could influence the way it was elongating. Alternatively, direction could be influenced by the substrate. Due to the size of fibrils and protofibrils that mostly exceeds the scanning area, it was difficult in this study to analyze their polarization.

In addition to the lateral association of protofibrils, the stacking of protofibrils and fibrils was also observed (Fig. 3B, Fig. 4 and Movie S2), a result consistent with two different heights observed in air. Interestingly, as suggested by real time imaging of their association shown in Movie S1 and in time lapse in Fig. 3B, protofibrils have a high propensity to interact with each other along their main axis via a zipper-like mechanism. Even rarely, elongation of a single protofibril over a fibril was also observed. In the time lapse in Fig. 4, a single protofibril with a 12 nm apparent diameter (grey arrowhead) is stacked on top of a fibril composed of 2 laterally associated protofibrils (see the profile shown in Fig. 4E). Real time imaging of the elongation of a protofibril at the top of a bundle of protofibrils is shown in Movie S2. During these experiments no more than two protofibrils or fibrils were stacked.

Structures suggesting branching of protofibrils or fibrils was also observed in liquid (white arrowhead in Fig. 2C and Movie S1) but HS-AFM experiments clearly indicated that these structure in fact corresponds to interaction between the extremities of growing protofibril or fibril with the side of another and not to formation of two protofibrils from a parental structure (Movie S1). These structures were nevertheless not so often observed as compared to experiments in air. This can be explained by the fact that AFM imaging in air was performed with mature lithostathine whereas this maturation was performed in the buffer bathing the tip with HS-AFM. In addition angle measured between the main axis of two protofibrils or fibrils was often around 70°. Also expected from results in air, overlapping of fibers was observed in liquid (Fig. 2C). This phenomenon increased as a function of time upon trypsin incubation and ultimately resulted in a complex network or tangle of fibrils (Fig. 2D). Overlapping most probably results from imaging a network of fibers in 2 dimensions.

**Figure 5:**
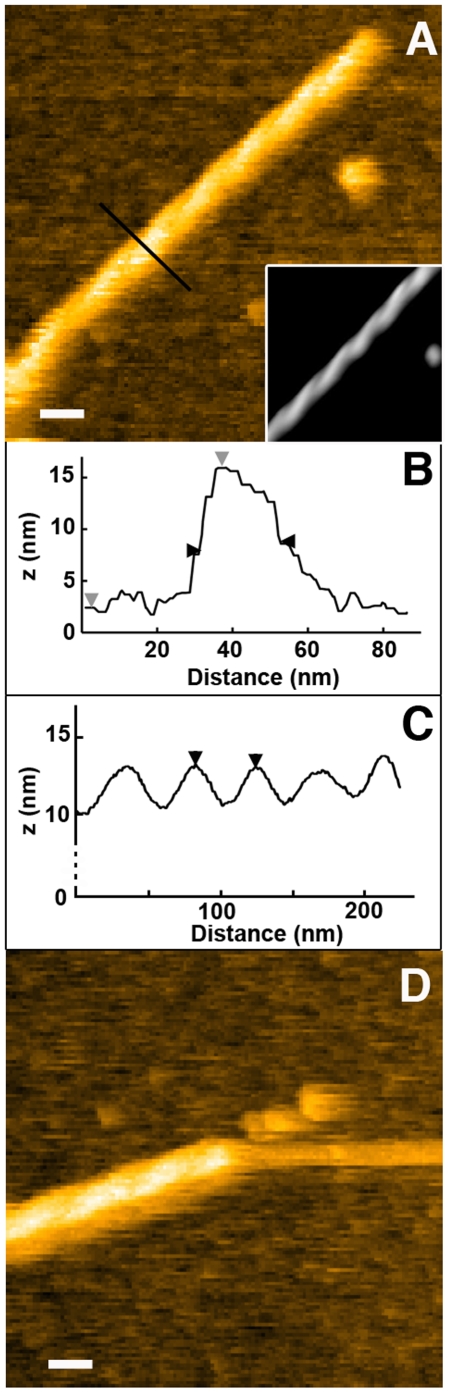
Height images of helical fibrils of lithostathine. A and D) Winding of two protofibrils can form helical fibrils (highlighted in the inset in A that corresponds to the filtered image). The edge of this helix can be blunt (A) but one of the protofibril composing this helical fibril can be lonely observed (D), protruding from the helix. B) Profile delineated in A by the black line. Vertical and horizontal distances are 13.6 nm between grey arrowheads and 20.1 nm between black arrowheads, respectively. C) Longitudinal profile of helical fibril shown in the A inset (the distance between the black arrowheads is 44.3 nm). The scale bar is 30 nm and the height scale automatically adjusted.

In addition to isolated protofibrils and bundles of laterally associated protofibrils, a new type of lithosthatine assemblies was observed using HS-AFM imaging in liquid, namely helical fibrils (Fig. 5 and Movie S3). These structures were formed from two left-handed winded protofibrils as supported by their apparent diameter of 24.2±0.8 nm as well as the height above the mica of 14.6±0.7 nm (Fig. 5B) (height of a single protofibril as compared to the mica is 7.8±1.7 nm). Helix periodicity was 52.7±11 nm ranging from 44 to 74 nm (Fig. 5C). Some helical fibrils appeared to be symmetric with blunt ends (Fig. 5A) whereas others had one of the protofibril longer than the other, forming a stiff protofibril (Fig. 5D). This observation suggests that the two protofibrils had different lengths or that their winding took place after their formation. Similar partial winding has also been observed in aggregating Aß peptide [30]. During our experiments, slight helical unwinding was sometimes observed (Movie S3) but we never imaged any winding process or any formation of such helical structure, strongly suggesting that helical fibril of lithostathine could only be formed in bulk without any interaction with mica. No higher order helical structures were observed (data not shown). It is important to differentiate these helical fibrils from helical protofibrils that have been described in a previous publication and correspond to stacking of lithostathine tetramers [26].

## Discussion

In this work we carried out HS-AFM experiments under physiological conditions allowing further structural characterization of protofibrils and fibrils of lithostathine, a protein involved in Alzheimer's disease, as well as real-time visualization of formation of these assemblies. Topography obtained using HS-AFM in liquid is in good agreement with that obtained in air with a classical setup or with EM experiments ([26] and Fig. S2) in terms of protofibril diameter and ability of protofibrils to laterally associate to form bundles plated on the substrate (Fig. 1). In addition, our data clearly indicate that one lithostathine protofibril can be stacked on top of another (we can exclude a sedimentation process since HS-AFM imaging was performed with the sample above the tip, see Materials and Methods section). Such behavior highlights the propensity of lithostathine protofibrils to associate along their main axis and real time observation elucidates the manner in which a protofibril associate (vertically or laterally) to another protofibril or to a fibril. The association propagated along their main axis (Fig. 3), strongly suggesting a zipper-like mechanism that could be related to the model of fibril formation through swapping domains proposed for lithostathine (see introduction and [25]). We can exclude that these bundles were induced by a strong interaction with the mica because this feature seems to be singular to a few amyloid-like proteins such as transthyretin and human calcitonin [31]–[33] and, to our knowledge, not observed with amyloid ß peptides. In addition similar organization was observed when lithostathine S1 form was deposited on highly ordered pyrolytic graphite and imaged with AFM [26] or on carbon-coated grid and observed with EM (see Fig. S2). It is noteworthy to mention that, in both EM and AFM in air experiments, the S1 form was formed in solution before the coating and therefore a random deposition of protofibrils could not generate such bundles.

We especially provide new insights into the dynamics of elongation of lithostathine protofibrils and fibrils on a relatively short time scale (1 image/s). Elongation process appears to be very rapid (a few tens of nanometers per sec). However it cannot explain the difference observed with what had been calculated from AFM experiments with amylin peptides (1.1 nm/min) [34] or with Aß1–40 protofibrils (1.3 nm/min) [35]. Interestingly it compares well with recent results obtained for the elongation of Aß25–35 fibrils on mica (ranging from 5 to 100 nm/s) using scanning-force kymography, a high temporal resolution method also performed using fibrils coated on mica [36]. So huge difference in the elongation process between different publications remains to be clarified but it is tempting to speculate that this phenomenon largely depends on the protein and on the molecular mechanism of its elongation. HS-AFM also indicates that lithostathine elongation seems to oscillate between two states, a blocked state where lithostathine elongation is frozen, and a processing state during which the velocity is roughly constant (see an example of the two states in Movie S1). At this time it is difficult to discriminate if the former is due to strong interaction with the substrate or if it really corresponds to native mechanism. The latter most probably corresponds to addition of pre-assembled lithostathine tetramers to protofibril end, but spatial resolution of our setup was certainly too low to visualize such details of assembly. Nonetheless, it should have been sufficient to observe addition of a pre-formed protofibrils to the end of another, and this was never observed. From a mechanistic point of view, the fact that protofibril elongation was only observed for long incubation time with trypsin suggests that a critical concentration of processed lithosthathine is required for the formation of tetramer or for tetramer annealing to the protofibril end.

The shape of lithostathine protofibrils and fibrils were mainly straight and very similar to the ura2p protein, a saccharomyces cerevisiae protein with prion properties [37], and to TTR_105–115_, a short peptide that serves as a model system for amyloid fibrils [32]. It could correspond to the type I of Aß fibrillar assemblies according to a recently published classification, namely straight and rigid fibers with a diameter of 10–15 nm [38]. Despite this apparent stiffness, lithostathine protofibrils could nevertheless twist to form helical fibrils, always composed of two paired protofibrils. Similar organization has been observed for several amyloid proteins such as ura2p mentioned above [39], Aß peptides, and α-synuclein [40] and it was suggested that winding/unwinding process of α-synuclein was essential for its growth process [30]. As described with Aß(1–40) peptides [30], [35], [41], pitch in paired helical filaments or axial cross-over spacing was variable indicating that lithostathine can have different forms even when incubated under the same conditions. Lithostathine helical structure also compares well with paired helical filaments described for the microtubule-associated protein Tau expressed in neurofibrillary tangles, an intracellular structure present in AD [42]. A nice example of AFM imaging of Tau in liquid has been recently published [43]. It is important to notice that no elongation of protofibrils was observed for helical structure of lithostathine. At the opposite, we have been able to observe elongation of straight protofibrils, even when they were interacting with another protofibril or fibril, laterally associated with an existing fibril or stacked on top of it (Figures 3 and 4). Similar behavior has been suggested for the Aß25–35 peptide [36]. Interestingly the velocity of elongation of a fiber stacked on top of another was in the same range as that observed for a fibril growing on the mica surface, also suggesting that lithostathine-mica interaction was loose under our experimental conditions. This interpretation is strengthened by the fact that lithostathine assemblies were sensitive to the tip scanning that sometimes swept away protofibrils or fibrils (data not shown).

Branching of Aß fibrils has been previously observed using AFM and EM and is proposed to be essential for the post-nucleation growth process [30], [44]. Similarly glucagon fibrils can generate new fibril ends by continuously branching into new fibrils [45]. In this case new fibrils mostly grew in the forward direction of the parent fibril with a preferential angle. AFM imaging in air (this study and [26]) suggested lithostathine protofibril branching but our HS-AFM experiments clearly indicate that these structures are different from those observed with glucagon and Aß proteins and was therefore generated by the end of elongating protofibrils or fibrils interacting with the edge of a second fibril. It did not correspond to formation of a new structure from a parent fiber. Because these structures were also observed using AFM in air and EM (Fig. S2) in which the different structures were formed in the bulk before coating on mica or on the grid, we could hypothesize that the elongated protofibril has a good affinity for the edge of preformed protofibrils. Interestingly, as observed with glucagon [45], a preferred angle was observed between two branched lithostathine fibers. At this time it is however difficult to elucidate a precise mechanism. It could be due to the fact that, when the angle is smaller than the preferred angle, two fibers have a tendency to laterally associate or to a preferential orientation of fibrils due to interaction with mica as previously described with amyloid proteins such as α-synuclein [46] or Aß peptide [47]. This point remains to be clarified.

Two distinct stages have been proposed for ß-amyloid fibrillization, namely nucleation and elongation (reviewed in [48]). Spherulitic structures with radial growth of fibrils have been observed with Aß(1–40) fibrils [38] and nuclei of Ure2p proteins have been identified as precursors of Ure2p fibrils [49]. Despite the fact that we have observed globular structures immobilized on mica that could correspond to nuclei of lithostathine tetramers, no nucleation areas were clearly identified. Such a result could be explained by the fact that, once in a growing state, elongation velocity is high and consequently the probability to observe such an event is low. It also strongly suggests a different mechanism of fibrillization for ß amyloid peptides as compared to lithostathine, since no spherulitic structures were observed using AFM in air or in liquid.

Taken together, our results provide new insights into lithostathine protofibril elongation and assembly. Lithostathine shares some features with Aß proteins such its ability to form helical structures but mechanism of lithostathine protofibrils formation appears to be different from that of Aß proteins or glucagon. It is important to mention that several properties of lithostathine could not have been determined using a longer timescale and HS-AFM thus appears as a powerful technique for the analysis of amyloid proteins.

## Methods

### Lithostathine Purification

Recombinant lithostathine was produced from Chinese ovary cells and purified on immunoaffinity column as described previously [24]. Protein samples were then frozen in liquid nitrogen and stored at −80°C until use.

### AFM imaging in air

AFM was performed in air with a Nanoscope IIIA (Veeco, Dourdan, France) using FMR Nanosensors or AC160 TS Olympus cantilevers. S1 form of lithostathine was coated in 15 mM Tris pH 7.5 at a concentration of 30 µg/ml on freshly cleaved mica (Goodfellow, Lille, France), rinsed with water, dried with nitrogen and kept in a dessicator until imaging. The scan rate was between 0.2 to 1 Hz.

### High speed AFM in liquid

The HS-AFM apparatus used in this study is basically the same as that previously reported by Ando et al [50]. Images were acquired at a 1 image/s rate using very sharp cantilevers with a 200 mN/m spring constant and a resonance frequency in water of 1.2 MHz (Olympus). Experiments were performed with intact protein at a concentration of 40 µg/ml that was then processed using a 1% trypsin solution, directly added in the AFM fluid cell, to generate the S1 form. Trypsin cleavage as well as AFM imaging was performed in 10 mM Tris, 100 mM NaCl, pH 8 buffer.

## Supporting Information

Figure S1 Elongation velocity of fibrillar lithostathine. The graph represents the growth distance of fibrils or protofibrils as a function of time. The zero values are origins of measurement in time and position. The bar errors correspond to S.D. values.
(0.05 MB TIF)

Figure S2 Electron microscopy micrographs of the lithostathine S1 form. The S1 form was coated on a Formvar-coated copper grid for 60 s, dried with a filter paper and stained with uranyl acetate as previously described (Gregoire C, et al. (2001) EMBO J 20: 3313–3321). Specimens were then observed with a Jeol 1220 transmission electron microscope. Lateral association of lithostathine fibrils was clearly observed in A and B. White arrowheads highlight overlapping of fibrils whereas the black arrowhead indicates the association of the end of two laterally associated protofibrils with the edge of another fibril. The scale bar is 250 nm.
(3.80 MB TIF)

Movie S1 High Speed AFM imaging of lithostathine. After 30 min trypsin incubation of the full-length lithostathine, fibrils as well as globular structures can be observed. The movie shows the growth of two different fibrils that are blocked by the edge of another fibril. In the last part of the movie, the association of one fibril on top of another is clearly observed. Most of the fibrils seem to be composed of several laterally associated protofibrils (see Fig. 2B). The scan size is 300 nm×300 nm (128×128 pixels) and the height scale is automatically adjusted during scanning. The scanning rate is 1 image/s and the movie is accelerated five times.
(10.38 MB AVI)

Movie S2 High Speed AFM imaging of lithostathine protofibril elongation stacked on the top of laterally associated protofibrils. The movie shows the growth of one protofibril at the top of a bundle of laterally associated protofibrils. The scan size is 300 nm×300 nm (128×128 pixels) and the height scale is automatically adjusted during scanning. The scanning rate is 1 image/s and the movie is accelerated five times.
(0.25 MB AVI)

Movie S3 High Speed AFM imaging of helical fibrils. Two sequences of helical fibril images are shown in this movie. First sequence: the slight helical unwinding is shown. A single protofibril is also swept away by the tip. Second sequence: real time imaging of one single protofibril protruding from a long paired helical filament. The coating of one protofibril on mica is also observed. The scan size is 300 nm×300 nm (128×128 pixels) and the height scale is automatically adjusted during scanning. The scanning rate is 1 image/s and the movie is accelerated five times.
(9.59 MB AVI)
