# Supplementary figures and images for: Deciphering the Structure, Growth and Assembly of Amyloid-Like Fibrils Using High-Speed Atomic Force Microscopy
Source: PLoS One. 2010 Oct 8;5(10):e13240. doi: 10.1371/journal.pone.0013240 (PMC2951901; doi:10.1371/journal.pone.0013240)

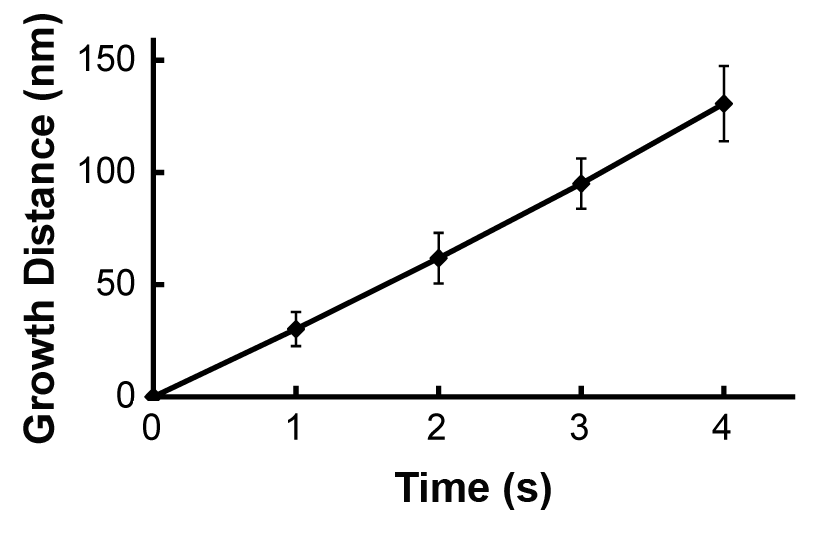

Supplement: Figure S1 — Elongation velocity of fibrillar lithostathine. The graph represents the growth distance of fibrils or protofibrils as a function of time. The zero values are origins of measurement in time and position. The bar errors correspond to S.D. values. (0.05 MB TIF) [file pone.0013240.s001.tif]

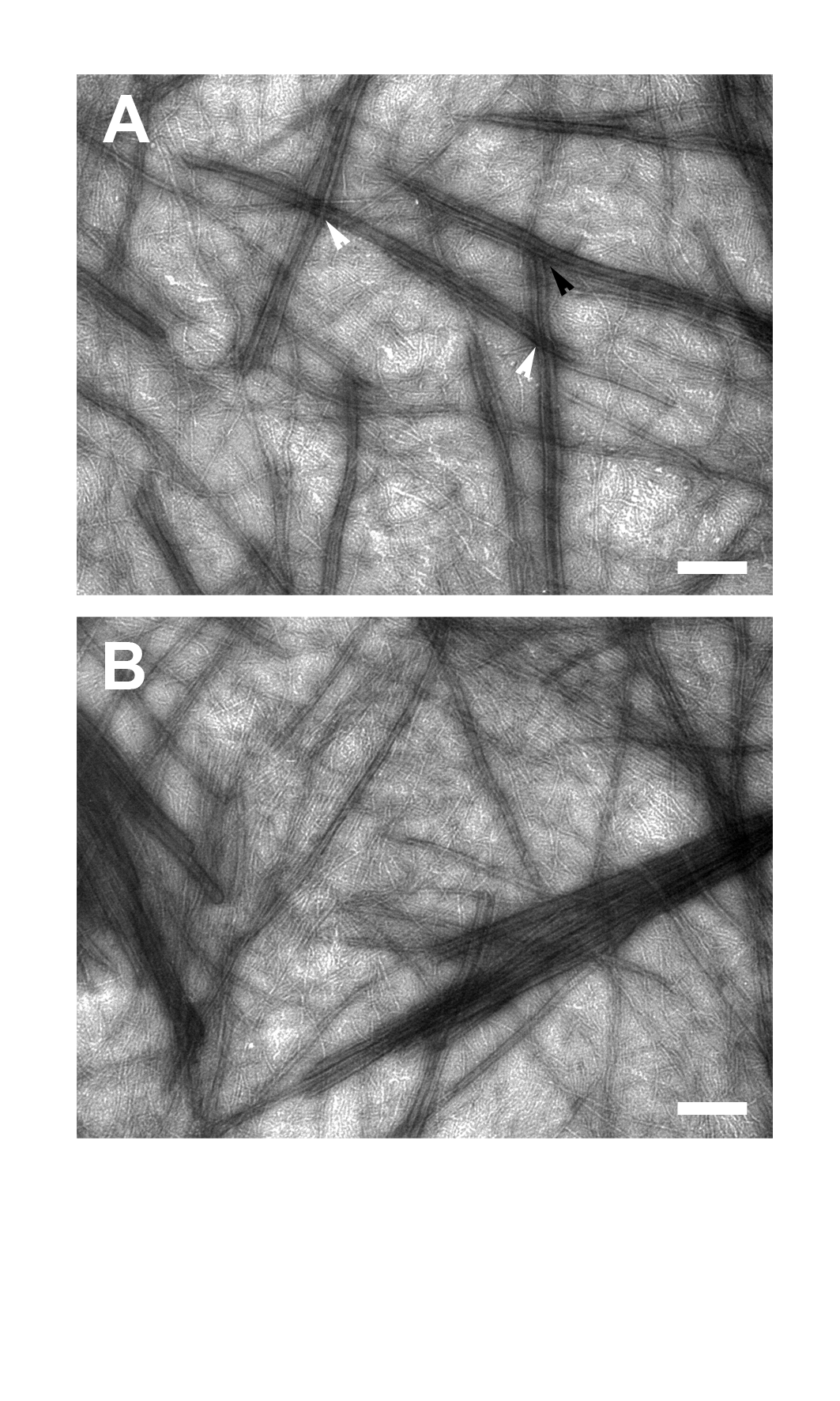

Supplement: Figure S2 — Electron microscopy micrographs of the lithostathine S1 form. The S1 form was coated on a Formvar-coated copper grid for 60 s, dried with a filter paper and stained with uranyl acetate as previously described (Gregoire C, et al. (2001) EMBO J 20: 3313–3321). Specimens were then observed with a Jeol 1220 transmission electron microscope. Lateral association of lithostathine fibrils was clearly observed in A and B. White arrowheads highlight overlapping of fibrils whereas the black arrowhead indicates the association of the end of two laterally associated protofibrils with the edge of another fibril. The scale bar is 250 nm. (3.80 MB TIF) [file pone.0013240.s002.tif]
